# Supplementary material for: Understanding the impact and causes of ‘failure to attend’ on continuity of care for patients with chronic conditions
Source: PLoS One. 2021 Mar 2;16(3):e0247914. doi: 10.1371/journal.pone.0247914 (PMC7924779; doi:10.1371/journal.pone.0247914)
Supplement: S1 Checklist — (DOCX) [file pone.0247914.s001.docx]

Consolidated criteria for reporting qualitative studies (COREQ): 32-item checklist

| Domain 1: Research team and reflexivity | Response (and manuscript line and page number) |
| --- | --- |
| Personal Characteristics |  |
| 1. Interviewer/facilitator. Which author/s conducted the interview or focus group? | Page 1  The authors of this paper are the researchers involved in the Nurse Navigator evaluation. Interviews were conducted by member of the evaluation research team, in particular authors a, 2, 3, 7 and 12.  Two researcher completed the initial coding, authors 1 and 3, with authors 1,2,3,4,5,6 and 7 partaking in coding and refining of themes. |
| 2. Credentials. What were the researcher's credentials? E.g. PhD, MD | Page 1  The credentials of the authors are as follows  **Amy-Louise Byrne** RN, BN, GCEmerg, GCCFH,  Associate lecturer, School of Nursing, Midwifery and Social Science  Central Queensland University  **Professor Desley Hegney** RN, BA (Hons) PhD  Research Division  Central Queensland University Brisbane Campus  School of Nursing, The University of Adelaide, South Australia  **Associate Professor Clare Harvey** RN, BA(Cur), MA, PhD  Associate Professor, School of Nursing, Midwifery and Social Sciences  Central Queensland University  **Dr Adele Baldwin** PhD, MNSt, GCETT, RN, RM  Senior Lecturer, School of Nursing, Midwifery and Social Sciences  Central Queensland University  **Professor Eileen Willis** PhD  Emeritus Professor, School of Nursing, Midwifery and Social Sciences  Central Queensland University  **David Heard**  Research Officer, School of Nursing, Midwifery and Social Science  Central Queensland University  **Professor Jenni Judd** DHSc, MPH, MEd, DipHPE  School of Health Medical and Applied Sciences  Central Queensland University  **Janine Palmer** RN, NP  Hawke’s Bay District Health Board  New Zealand  **Dr Janie Brown** BN, MEd, PhD  Associate Professor, School of Nursing, Midwifery and Paramedicine  Curtin University  **Dr Brody Heritage** BPsych (Hons), PhD  College of Science, Health, Engineering and Education  Murdoch University  **Dr. Shona Thompson** PhD  Eastern Institute of Technology  New Zealand  **Bridget Ferguson** RN, RM,  BNurs, GradCertMid, MMID, MPH.  Lecturer, School of Nursing, Midwifery and Social Sciences  Central Queensland University |
| 3. Occupation. What was their occupation at the time of the study? | See above |
| 4. Gender. Was the researcher male or female? | Page 1  The research team is made up of a gender diverse team. |
| 5. Experience and training. What experience or training did the researcher have? | Page 1  Provided within the affiliations and qualifications above |
| Relationship with participants |  |
| 6. Relationship established. Was a relationship established prior to study commencement? | Page 7  The research is part of a wider study evaluating the Nurse Navigator Service. The relationship between researchers and subjects is a professional collaborative one, and one which is ongoing in nature. |
| 7. Participant knowledge of the interviewer  What did the participants know about the researcher? e.g. personal goals, reasons for doing the research | Page 7  Participants are known to researcher and visa versa, as this research is part of a wider evaluation. Additionally, the evaluation applies a theory of change methodology to allow for emergent themes to be explored and adopted by participants. |
| 8. Interviewer characteristics. What characteristics were reported about the interviewer/facilitator? e.g. Bias, assumptions, reasons and interests in the research topic | Page 7  The phenomenon of FTA was an emergent theme of the Nurse Navigator evaluation, thus became the subject of analysis organically. Data was collated and independently analysed by two members of the research team and then discussed with seven other researchers to ensure bias and assumptions were adequately addressed. |

| Domain 2: study design |  |
| --- | --- |
| Theoretical framework |  |
| 9. Methodological orientation and Theory. What methodological orientation was stated to underpin the study? e.g. grounded theory, discourse analysis, ethnography, phenomenology, content analysis | Page 9 and 10  The Nurse Navigator evaluation uses a mixed method, theory of change framework to explore the services and to analyse emergent themes.  This paper a thematic analysis of qualitative data |
| Participant selection |  |
| 10. Sampling. How were participants selected? e.g. purposive, convenience, consecutive, snowball | Page 7 and 9  All Nurse Navigators across Queensland Health are invited to participate in this wider study. As part of the evaluation study, navigators provide vignettes, case studies and partake in semi-structured interviews and focus groups. The data collected from navigators was analysed with the lens of FTA for this research paper. |
| 11. Method of approach. How were participants approached? e.g. face-to-face, telephone, mail, email | Page 7  Interviews were conducted fact to face, via teleconferencing. |
| 12. Sample size. How many participants were in the study? | Page 8  We have reported on the quantitative data showing no change in FTA count. The sample size is 52. |
| 13. Non-participation. How many people refused to participate or dropped out? Reasons? | N/A |
| Setting |  |
| 14. Setting of data collection. Where was the data collected? e.g. home, clinic, workplace | Page 7 and 8  Interviews were collected in a work and home settings, via the mediums described above. For patient interviews, these were conducted in a place decided by the patient, home, community. |
| 15. Presence of non-participants. Was anyone else present besides the participants and researchers? | N/A |
| 16. Description of sample. What are the important characteristics of the sample? e.g. demographic data, date | Page 4  The demographic of nurse navigators and their patients is described throughout the paper. Patients are complex, with multiple chronic conditions. NN are senior registered nurses working for Queensland Health |
| Data collection |  |
| 17. Interview guide. Were questions, prompts, guides provided by the authors? Was it pilot tested? | N/A |
| 18. Repeat interviews. Were repeat interviews carried out? If yes, how many? | N/A |
| 19. Audio/visual recording. Did the research use audio or visual recording to collect the data? | Page 8  Teleconferencing meetings were conducted via Zoom or Teams. |
| 20. Field notes. Were field notes made during and/or after the interview or focus group? | N/A |
| 21. Duration. What was the duration of the interviews or focus group? | Interviews were conducted for approximately 1 hour |
| 22. Data saturation. Was data saturation discussed? | N/A  The NN evaluation is ongoing. This research report on an emergent theme of the evaluation. |
| 23. Transcripts returned. Were transcripts returned to participants for comment and/or correction? | No, however the research evaluation is ongoing. |

| Domain 3: analysis and findings |  |
| --- | --- |
| Data analysis |  |
| 24. Number of data coders.  How many data coders coded the data? | Page 10  Coding was completed by 7 members of the research team. |
| 25. Description of the coding tree. Did authors provide a description of the coding tree? | N/A |
| 26. Derivation of themes. Were themes identified in advance or derived from the data? | Page 10 and 11  Themes were dev\rived from the data |
| 27. Software. What software, if applicable, was used to manage the data? | N/A |
| 28. Participant checking. Did participants provide feedback on the findings? | N/A |
| Reporting |  |
| 29. Quotations presented. Were participant quotations presented to illustrate the themes / findings? Was each quotation identified? e.g. participant number | Page 8, 11- 20  The research predominantly uses quotes from Nurse Navigators, however where possible, patient quotes were included to triangulate data |
| 30. Data and findings consistent. Was there consistency between the data presented and the findings? | Page 20 |
| 31. Clarity of major themes. Were major themes clearly presented in the findings? | Page 11-20  Major themes were present in the findings and explored in the discussion. |
| 32. Clarity of minor themes. Is there a description of diverse cases or discussion of minor themes? | N/A |
