# Supplementary material for: Understanding the impact and causes of ‘failure to attend’ on continuity of care for patients with chronic conditions
Source: PLoS One. 2021 Mar 2;16(3):e0247914. doi: 10.1371/journal.pone.0247914 (PMC7924779; doi:10.1371/journal.pone.0247914)
Supplement: Attachment — Submitted filename: renamed_2a620.docx [file pone.0247914.s002.docx]

Reviewer: Stefan Nöst

PONE-D-20-11849

The aim of the presented study is to capture the reasons and define the concept of “Failure to Attend” from the perspectives of patients and nurse navigators. Nurse navigators ensure continuity of care of individual patients with multiple and complex chronical conditions by improving case and care management and this include facilitating self-management and supporting compliance of the patients, with the aim to reduce preventable hospital admissions and improve patient outcomes. With regard to this, FTA is defined as an indicator of hospital utilization. This study is part of a broader interim evaluation of the nurse navigator service and therefore the authors secondary analyse interview data with the thematic focus on FTA.

The authors explore the phenomenon “Failure to Attend” which is very relevant concerning to patients’ access to and continuity of care and the study also make a contribution to the actual discussion of inappropriate language used in care setting (e.g. discussion of “compliance”). But they implicitly also describe another phenomenon: The difference between Talk and Action in Health Care Settings which helps to reduce complexity.

Despite that, some shortcomings exist and the paper has to be strengthen before publication to improve quality. Therefore, some suggestions and recommendations are following:

- Introduction/Background -

The focus is more on the project setting and context but I recommend to take also the evidence of the used theoretical concepts into account (e.g. Continuity of Care and its associations to nursing case management or nursing led transition management; e.g. state of the research onto FTA)

- Aim -

The authors provide divergent phrasing of the aim:

“The aim of this paper is to capture the reasons for ‘Failure to Attend’ (FTA) through the narrative of nurse navigators and their patients and to explore how it is defined by the patient.” (32/33) (thematic coding/content analysis is appropriate)

“The aim of this paper is to provide an understanding of the concept, use and impact of the phrase ‘Failure to Attend’ on the relationship between health services and navigated patients.” (140/141) (a deeper, more interpretative method would be appropriate)

This is not only a divergence in wording but rather in the appropriateness of the methods used for data analysis (please see the hints within the brackets).

In my view, there is no clear fit between the different aims, the research questions, the methods used and the reported results. Thus I recommend to adapt the divergent aims and rephrase it consistently, in reference to the methods used. In that regard I also recommend to customize the research questions whilst taking the results into account.

- Methodological issues -

It is not clear how each of the 15 authors contributed to the study or this paper. Regarding this, the COREQ-checklist is also fulfilled in an insufficient way and the given information did not create transparency. (e.g. Who conducted the group and individual interviews? Data were collected “…by members of the research team”. Is the research team of this study identical as the team who conducted the evaluation?). I recommend to clarify the contribution of the authors and take the COREQ guideline into account in reporting this qualitative research.

The reported research design describes the interim evaluation of the nurse navigators approach. Interviews were conducted as part of this evaluation and the transcribed interviews were analysed with the thematic focus on FTA (“the management of FTAs and the effects this label has on patients when they are unable to attend specialist outpatient appointments.” (170/171)). It is unclear if FTA was explicitly included into the data collection (e.g. as theme within the interview guide) in an iterative way or if primarily collected data of the nurse navigator evaluation were analysed with the new lens of FTA? I recommend to clear the difference explicitly if it was an explanatory sequential mixed-method design including FTA as theme primarily OR a secondary analyses of the qualitative evaluation data? I think it was the second one, but in my view the manuscript is ambiguous with regard to that.

Data analyses: A combined deductive inductive approach was used to analyse data and out of this procedure a code system evolved. I recommend to report the code system (e.g. as mind-map, code tree or table) to ensure transparency and to guide the reader through the results by visualization.

Description of data analysis is a bit confusing: “Second cycle coding was then completed by the research team members collaboratively, and codes were … further developed within the analytic lens of the nurse navigator service” (208-210). Why did the authors not use the lens of FTA?

- Ethics -

I recommend to explicitly mention the research ethics committee, not only the reference number.

- Results -

Conflicting patients’/health services priorities.

In my view, the authors report predominantly reasons of failure and they identified access barriers (e.g. money, time, distance, disability, equipping). I cannot comprehend the label of this major code “Conflicting patients’/health services priorities”. I recommend to provide a definition or meaning of the major code labels to clarify this.

Failure to Recognise Personal Stigma of FTA.

Only narratives of nurse navigators were quoted. Is this in line with the aims and research questions of the study?

Bridging the gap.

Nurse navigator’s perspectives were interpreted as patient centred by nursing researcher and based on this interpretation this was reasoned as bridging behaviour. I recommend to reconsider this section and keep the difference in talk vs. action (Brunson / Olson 1993) into account. I also recommend to frame this result explicitly as nurse perspective.

- Discussion/Conclusion -

The authors offer a successful discussion and adequate conclusion. They discuss “Failure to attend” in contrast with other concepts, e.g. "compliance", and they argue to differ the phrase “Failure to attend” from the phrase “Appointment did not proceed” and the last one should be used when referring to a patient’s inability to attend an appointment
