# Supplementary material for: Understanding the impact and causes of ‘failure to attend’ on continuity of care for patients with chronic conditions
Source: PLoS One. 2021 Mar 2;16(3):e0247914. doi: 10.1371/journal.pone.0247914 (PMC7924779; doi:10.1371/journal.pone.0247914)
Supplement: Attachment — Submitted filename: Response to Reviewers.docx [file pone.0247914.s005.docx]

PLOS ONE	20/01/2021

To the Editor

It is with great pleasure that we submit for review *Understanding the impact and causes of ‘failure to attend’ on continuity of care for patients with chronic conditions.*

This thematic analysis was conducted to better under the impacts that the phenomenon of ‘Failure to Attend’ has on people with multi, complex chronic conditions. **Themes emerging from the data were: conflicting patient/health service priorities; failure to recognise personal stigma of FTA; and** bridging the gap. This article advocates for further consideration of collaborative models that engage the patient in their care journey and for consideration of the language used within the outpatient acute hospital setting, proposing the term ‘appointment did not proceed.’

This is the fourth submission of this manuscript to PLOS one with revisions now available below. Please see response to reviewers below.

Please note: We note that some of the changes suggestion in this latest round are the same as the previous round which we believe have been adequately addressed.

The authors declare no conflict of interest.

Funding (financial support) for this project was provided by Queensland Health. The funders had no role in study design, data collection and analysis, decision to publish, or preparation of the manuscript. No other funding was received for the project. No authors have received a salary from the funders.

Yours Sincerely

Amy-Louise Byrne, corresponding author

**Third Revisions**

| **Reviewer Feedback** | **Authors amendments and comments** |
| --- | --- |
| Regarding to my first review: the COREQ-checklist is further fulfilled in an insufficient way: First, I cannot identify your responded information within the manuscript for the COREQ questions 2, 3, 5. Second, your response to question 8 is not suitable. Third, question 8 refer to recording (possible response: "interviews are recorded and transcribed by a professional transcription service"). | Thank you for the detailed review and feedback.  The Manuscript has now been improved to include your suggestions. Plos One does not include the qualifications of authors in the title so we have amended the manuscript to read;  COREQ 2- Initial coding was independently completed by two members of the research team (author one is a PhD candidate and author two holds a PhD) who coded the data by single word, paragraph and full content (14). Second cycle coding was then completed by seven research team members collaboratively, all of whom hold a PhD qualification, and codes were reconfigured, recategorized, resynthesized and further developed within the analytic lens of FTA (16).  COREQ- 3- The university affiliations are listed on the title page. PloS One formatting does not include positions, or titles in the cover page. I have added these to the COREQ checklist to maximise transparency  COREQ 5- As above  COREQ 8- This has been amended to read; The phenomenon of FTA was an emergent theme of the Nurse Navigator evaluation, thus became the subject of analysis organically. Data was collated and independently analysed by two members of the research team and then discussed with seven other researchers to ensure bias and assumptions were adequately addressed. |
| Regarding to my first and second review: the visualisation of the results (codes and/or themes) would be very helpful for reader guidance. | This has been addressed in the previous round of feedback.  The manuscript reads.  A coding tree is not available for this data.  While questions around FTA had not been specifically asked in interviews, the topic came up in several interviews, particularly with the nurse navigators because it was central to the their work and they saw it as part of the explanation for patient’s alienation from the system. Initial coding was independently completed by two members of the research team (author one is a PhD candidate and author three holds a PhD) who coded the data by single word, paragraph and full content (14). Second cycle coding was then completed by seven research team members collaboratively, all of whom hold a PhD qualification, and codes were reconfigured, recategorized, resynthesized and further developed within the analytic lens of FTA (16). Final consensus of themes was achieved collaboratively within the research team with collated themes collapsed into major themes with accompanying quotes to demonstrate the themes (See S1 Fig COREQ Checklist). The majority of quotes come from the nurse navigators, but where possible we have added patient comments as a form of triangulation. |
| Regarding to my second review: to demonstrate variety of the reported quotes, I suggest using pseudonymized references (e.g. Nurse Navigator 1 vs. Nurse Navigators 8) to ensure transparency. | This has been addressed in the second review.  For consistency, this was changed to Nurse navigator throughout the entire document.  In order to keep the experiences clear, we have decided to stay with roles in order to highlight the lived experiences of those in these roles. |
| In addition to my second review: To characterize the sample usage of descriptive statistics are usual to ensure research transparency. | We believe that the research design addresses all requirements for research credibility, validity, and rigor. |

**Second Revisions**

| **Reviewer feedback** | **Authors amendments and comments** |
| --- | --- |
| Thank you for reviewing this manuscript a second time. The authors processed my suggestions for the most part which now led to an improved and sharpened manuscript, but some minor shortcomings remained | Thank you for the revisions and further feedback. Please see further amendments below. |
| From a methodological view Ich recommend specify the sample size (counts of conducted nurse navigator interviews as well as patient interviews) of the primary qualitative study which were now secondary analysed. | This has now been amended and reads  ‘To date, 25 individual and 14 group interviews have been completed with NNs. These interviews included NN across all areas of Queensland and are inclusive of special interest groups including a rural and remote group, midwifery group, aged care group and nurse navigators/practitioners’ group. Additionally, 49 interviews with consented NN patients were conducted across all areas of Queensland. This includes patients across all life stages (from paediatrics to aged care), all of whom suffer from chronic complex conditions and frailty and are under the care of the NN.’ |
| With regard to this, I also suggest to describe the sample characteristics at a glance, within the result section. | This has been addressed above. |
| Different terminology was used to cite the quotes: "Nurse Navigator", "nurse navigator" and "Navigator"). I recommend to harmonize this and with regard to transparency and in order to demonstrate variety within the sampling, I also suggest to use pseudonyms and not only roles to cite the quotes . | For consistency this has been changed to Nurse navigator throughout the entire document.  In order to keep the experiences clear, we have decided to stay with roles in order to highlight the lived experiences of those in these roles. |
| As already mentioned in the first review I suggest to visualise the categorization/code system with the aim to maintain and guide the reader. | A coding tree is not available for this data.  Coding has been described in the manuscript and reads  “While questions around FTA had not been specifically asked in interview, the topic came up in several interviews, particularly with the nurse navigators because it was central to the their work and they saw it as part of the explanation for patient’s alienation from the system. Initial coding was independently completed by two members of the research team who coded the data by single word, paragraph and full content (14). Second cycle coding was then completed by seven research team members collaboratively, and codes were reconfigured, recategorized, resynthesized and further developed within the analytic lens of FTA (14). Final consensus of themes was achieved collaboratively within the research team with collated themes collapsed into major themes with accompanying quotes to demonstrate the themes (See S1 Fig COREQ Checklist).” |

**First Revisions**

| **Reviewer feedback** | **Author response and changes** |
| --- | --- |
| Please amend your current ethics statement to include the full name of the ethics committee/institutional review board(s) that approved your specific study.  Once you have amended this/these statement(s) in the Methods section of the manuscript, please add the same text to the “Ethics Statement” field of the submission form (via “Edit Submission”). | Amended in system |
| We note that you have provided funding information that is not currently declared in your Funding Statement. However, funding information should not appear in the Acknowledgments section or other areas of your manuscript. We will only publish funding information present in the Funding Statement section of the online submission form.  Please remove any funding-related text from the manuscript and let us know how you would like to update your Funding Statement. Currently, your Funding Statement reads as follows:   [The funders had no role in study design, data collection and analysis, decision to publish, or preparation of the manuscript.] | Amended |
| Please include captions for your Supporting Information files at the end of your manuscript, and update any in-text citations to match accordingly. | Supporting information (COREQ Checklist) now listed at bottom of manuscript and cited in document p. 8. |
| **Reviewer One** |  |
| - Introduction/Background - The focus is more on the project setting and context but I recommend to take also the evidence of the used theoretical concepts into account (e.g. Continuity of Care and its associations to nursing case management or nursing led transition management; e.g. state of the research onto FTA) | Thank you for your comments. The introduction and background have been amended accordingly. |
| - Aim - The authors provide divergent phrasing of the aim:  “The aim of this paper is to capture the reasons for ‘Failure to Attend’ (FTA) through the narrative of nurse navigators and their patients and to explore how it is defined by the patient.” (32/33) (thematic coding/content analysis is appropriate)  “The aim of this paper is to provide an understanding of the concept, use and impact of the phrase ‘Failure to Attend’ on the relationship between health services and navigated patients.” (140/141) (a deeper, more interpretative method would be appropriate)  This is not only a divergence in wording but rather in the appropriateness of the methods used for data analysis (please see the hints within the brackets).  In my view, there is no good fit between the different aims, the research questions, the methods used and the reported results. Thus, I recommend to adapt the divergent aims and rephrase it consistently, in reference to the methods used. In that regard I also recommend to customize the research questions whilst taking the results into account. | Thank you for highlighting this. The aims have been changed to more accurately reflect the paper’s content and to ensure consistency between the abstract and the body of work.  This is now consistent between the abstract and body and reads  Abstract- “To understand the impact and causes of ‘Failure to Attend’ (FTA) labelling, of patients with chronic conditions.” P. 2  Body- “The aim of the reanalysis was to understand the impact and causes of ‘Failure to Attend’ (FTA) labelling of patients with chronic conditions” p. 6 |
| - Methodological issues - It is not clear how each of the 15 authors contributed to the study or this paper. Regarding this, the COREQ-checklist is also fulfilled in an insufficient way and the given information did not create transparency. (e.g. Who conducted the group and individual interviews? Data were collected “…by members of the research team”. Is the research team of this study identical as the team who conducted the evaluation?). I recommend to clarify the contribution of the authors and take the COREQ guideline into account in reporting this qualitative research. | Apologies for the lack of transparency. The COREQ has now been completed in detail, explicitly describing the researcher’s relationship to the method. This has been discussed in the method section. P. 7-8  COREQ is now listed as a supporting figure and cited in the manuscript. P.8. |
| The reported research design describes the interim evaluation of the nurse navigators approach. Interviews were conducted as part of this evaluation and the transcribed interviews were analysed with the thematic focus on FTA (“the management of FTAs and the effects this label has on patients when they are unable to attend specialist outpatient appointments.” (170/171)). It is unclear if FTA was explicitly included into the data collection (e.g. as theme within the interview guide) in an iterative way or if primarily collected data of the nurse navigator evaluation were analysed with the new lens of FTA? I recommend to clear the difference explicitly if it was an explanatory sequential mixed-method design including FTA as theme primarily OR a secondary analyses of the qualitative evaluation data? I think it was the second one, but in my view the manuscript is ambigous with regard to that. | This section of the paper has been amended to clearly show that the FTA focus was as a result of the initial data analysis in the overall study.  “The impetus to explore the FTA phenomenon qualitatively was based on the interim quantitative data analysis. Subsequently, during initial qualitative data analysis the term FTAs became apparent. The emergence of FTA as a phenomenon in both data sets, prompted a reanalysis of the qualitative data to focus on more specifically on FTAs.” P. 6 |
| Data analyses: A combined deductive inductive approach was used to analyse data and out of this procedure a code system evolved. I recommend to report the code system (e.g. as mind-map, code tree or table) to ensure transparency and to guide the reader through the results by visualization. Description of data analysis is a bit confusing: “Second cycle coding was then completed by the research team members collaboratively, and codes were … further developed within the analytic lens of the nurse navigator service” (208-210). Why did the authors not use the lens of FTA? | Thank you for the feedback. The method now reads  “While questions around FTA had not been specifically asked in interview, the topic came up in several interviews, particularly with the nurse navigators because it was central to the their work and they saw it as part of the explanation for patient’s alienation from the system. Initial coding was independently completed by two members of the research team who coded the data by single word, paragraph and full content (14). Second cycle coding was then completed by seven research team members collaboratively, and codes were reconfigured, recategorized, resynthesized and further developed within the analytic lens of FTA (14). Final consensus of themes was achieved collaboratively within the research team with collated themes collapsed into major themes with accompanying quotes to demonstrate the themes (See S1 Fig COREQ Checklist).” |
| - Results -  Conflicting patients’/health services priorities. In my view, the authors report predominantly reasons of failure and they identified access barriers (e.g. money, time, distance, disability, equipping). I cannot comprehend the label of this major code “Conflicting patients’/health services priorities”. I recommend to provide a definition or meaning of the major code labels to clarify this. | Thank you for your comments. The presentation of the themes has been amended to more clearly describe the themes and the supporting participant quotes.  These now read as;  Access Barriers  Failure to recognise personal stigma of FTA  Bridging the gap |
| Failure to Recognise Personal Stigma of FTA. Only narratives of nurse navigators were quoted. Is this in line with the aims and research questions of the study? | The majority of data collected on this subject were from Nurse Navigators. We have added the following sentence to clarify.  “The majority of quotes come from the Nurse Navigators, but where possible we have added patient comments as a form of triangulation.” P. 8 |
| Bridging the gap. Nurse navigator’s perspectives were interpreted as patient centred by nursing researcher and based on this interpretation this was reasoned as bridging behaviour. I recommend to reconsider this section and keep the difference in talk vs. action (Brunson / Olson 1993) into account. I also recommend to frame this result explicitly as nurse perspective. | Thank you for the suggestion. We have used the interpretation of Person-centred care as it is a key role principle of the Nurse Navigator service. We have amended this section to make this point clear and to make it clear that this is a nurse perspective. This now reads    “The first key role principle of the Nurse Navigator service is to coordinate person-centred care (1), thus a strong theme emerged within the nursing narrative about the role they play in delivering care to individuals. The nurse navigator narratives demonstrated the important role they play in bridging the gap between medical, allied health, community and acute services. They consistently spoke of how they helped a patient to change, align or reschedule appointments, describing how difficult it was for patients to coordinate multiple appointments across numerous services, geographically distant from each other. Their role is one of being an advocate for patients, bridging the gap through information sharing and discussion with service providers alongside, and on behalf of, their patients.” P. 15 |
| - Discussion/Conclusion - The authors offer a successful discussion and adequate conclusion. They discuss “Failure to attend” in contrast with other concepts, e.g. "compliance", and they argue to differ the phrase “Failure to attend” from the phrase “Appointment did not proceed” and the last one should be used when referring to a patient’s inability to attend an appointment | Your time and feedback is very much appreciated. |
| **Reviewer two** |  |
| Manuscript purpose to capture reasons for "failure to attend" is not truly satisfied through your research questions - How is the phrase FTA described? Priorities of providers that impact patients capacity to attend? and Impact of the FTA label? These are important research questions and should be reflected in the manuscript purpose. | Thank you for the comment. We have amended the questions and they now read   1. How do the nurse navigators and their patients use the term FTA? 2. What are the factors that lead to patients’ failure to attend? 3. What is the reported impact of being labelled on the patients’ wellbeing? |
| A few minor grammatical issues - example line 565 | Grammatical issues amended through out the manuscript. |
